# Did “*Kayoinoba*” Prevent the Decline of Mental and Physical Functions and Frailty for the Home-Based Elderly during the COVID-19 Pandemic?

**DOI:** 10.3390/ijerph18189502

**Published:** 2021-09-09

**Authors:** Mio Kitamura, Takaharu Goto, Shinji Fujiwara, Yasuhiko Shirayama

**Affiliations:** 1 Department of Oral Health Science and Social Welfare, Graduate School of Oral Sciences, Tokushima University, 3-18-15, Kuramoto-cho, Tokushima 770-8504, Japan; kumazaki1276@gmail.com; 2 Department of Prosthodontics and Oral Rehabilitation, Institute of Biomedical Sciences, Graduate School, Tokushima University, 3-18-15, Kuramoto-cho, Tokushima 770-8504, Japan; tak510@tokushima-u.ac.jp; 3 Mima City Koyadaira Municipal Medical Clinic, 295, Kawai, Koyadaira, Tokushima 777-0302, Japan; syfujiwara@jichi.ac.jp; 4 Department of Oral Health Science and Social Welfare, Institute of Biomedical Sciences, Graduate School, Tokushima University, 3-18-15, Kuramoto-cho, Tokushima 770-8504, Japan

**Keywords:** *Kayoinoba*, COVID-19, Kihon Checklist (KCL), frailty

## Abstract

The purpose of this study is to grasp the management situation of “*Kayoinoba*” under the conditions of self-quarantine due to the COVID-19 pandemic. It is also to clarify the efficacy of “*Kayoinoba*” using the Kihon Checklist (KCL) for the assessment of mental and physical functions in the elderly. The respondents were 136 elderly people aged 65 years and over who lived in A City, a standard rural area in Japan. The age, gender, living style, affluence for living, and the frequency of participation in “*Kayoinoba*” were examined by using the KCL as a self-completed questionnaire. Finally, 101 respondents were included in the final analysis. There was no difference in the participation status before and after the spread of COVID-19. The frailty ratio tended to decrease from 23.8% to 19.8% between the two periods, but there was no difference in the frailty ratio. It is suggested that the participants in “*Kayoinoba*” may have suppressed the deterioration of mental and physical conditions, excluding physical activity. This would prevent the frailty of the elderly, even during self-quarantine due to the spread of COVID-19.

## 1. Introduction

The new Coronavirus (severe acute respiratory syndrome Coronavirus 2, SARS-CoV-2) has continued to spread worldwide since the World Health Organization declared it a pandemic in March 2020. Its conclusion is presently unpredictable. In the prevention, screening, and treatment of COVID-19, the developments of medical internet of things-based healthcare systems, wearable biometric sensors, and real-time clinical monitoring are being promoted worldwide [1,2,3]. On the other hand, it has been pointed out that the spread of COVID-19 affects the mental health of the elderly and causes depression due to the development of anxiety [4,5,6]. In addition, more women experience increased depressive moods, anxiety, and loneliness compared with men, and there are concerns about increased pessimistic emotions and sleep disorders [4]. In addition, psychological investigations on irritability, confusion, anger, and frustration show that they increase the risk of family problems and make them more apparent, and may also increase the future risk of domestic violence, marital murder, suicide, and child abuse [6]. As mentioned above, COVID-19 has various influences on people’s lives. It is suggested that COVID-19 is likely to be more severe in the elderly and in those with underlying diseases. The Japanese government has therefore requested “self-quarantining” that involves people staying in their own homes. There is no legal basis for a lockdown in Japan. A self-quarantine, in which people voluntarily avoid going out for unnecessary and non-urgent matters, is expected to have a deterring effect on viral transmission. Conversely, self-quarantine lowers the activity of the elderly [7,8,9]. It may have a negative effect on their physical and mental health. Japan ranks first globally in the percentage of elderly people (65 years and older) at 28.7% [10].

In a super-aged society, extending a healthy life expectancy is an important issue. The highest priority is to reduce the medical and long-term care costs for the elderly generation and to reduce the tax and insurance burdens on the younger generations. The concept of frailty has been focused on when reviewing the extension of one’s healthy life expectancy [11,12]. Frailty is an intermediate state between a healthy individual and the need for nursing care that may return the person to a healthy state, with appropriate interventions. It is composed of three elements: physical, mental/psychological, and social frailties. Murayama et al. [13] and Shimada [14] reported that the ratios of the Japanese frail elderly who are 65 years of age or older were 8.7% and 11.3%, respectively.

**Figure 1:**
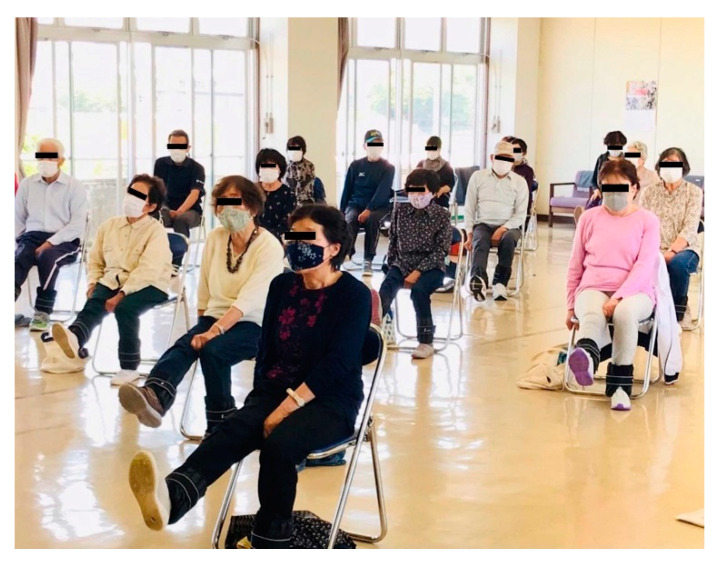
The elderly participate in the “*Kayoinoba*” in the A City Community Center.

As an early intervention for frailty in Japan, elderly people are encouraged to participate in “*Kayoinoba*” (Figure 1). “*Kayoinoba*” is one of the systems established to solve the “2025 issue”: An estimated one out of every three people will be over 65 years old, and one out of every five people will be over 75 years old in Japan in 2025. This is a long-term care prevention club that elderly residents can run voluntarily [15].

“*Kayoinoba*” is held in a public hall operated by the municipality. Various programs, such as physical and cognitive exercises, are proposed and implemented. Health counseling is regularly performed by public health nurses. However, when the COVID-19 pandemic spread, the public hall was closed and “*Kayoinoba*’s” activities were temporarily stopped. “*Kayoinoba*” was resumed almost immediately and has been intermittently active to this day owing to the following countermeasures: infection control, the shortening of time, and changes to the program’s contents. Although the efficacy of “*Kayoinoba*” has been validated in a conventional situation [16], it is unclear whether “*Kayoinoba*” has contributed to the maintenance of mental and physical functions. In addition, it is unclear whether it prevents frailty in the elderly during the COVID-19 epidemic and self-quarantine.

The study’s purpose is to understand the management situation of “*Kayoinoba*,” a health maintenance system for the elderly in Japan. It also aims to clarify the efficacy of “*Kayoinoba*,” by verifying the mental and physical functions of the elderly before and after self-quarantine due to COVID-19.

## 2. Materials and Methods

### 2.1. Respondents

The respondents were 136 home-based elderly people aged 65 years and over, who have lived in “A City” (73,431 people), Tokushima Prefecture and used “*Kayoinoba*” at least once a month. The aging rate of A City was 33.2% in February 2021. The respondents were selected by snowball sampling of the public health nurses belonging to the community general’s support center (CGSC). At the time of the survey, there were 137 “*Kayoinoba*” in A City, and the respondents were extracted from 14 of them.

The CGSC, which supports the elderly in the community, is provided by long-term care insurance in Japan. Public health nurses, social workers, and chief care managers have been assigned to the CGSC. They have worked for the health and long-term care of the elderly, in cooperation with the related organizations of medical care and public health. A City has seven CGSCs, and this survey was supported by all the CGSCs.

### 2.2. Operation of “Kayoinoba” under Self-Quarantine, Due to the Spread of COVID-19

“*Kayoinoba*” consists of the following four conditions: (1) The purpose is to prevent long-term care. It involves activities, such as gymnastics and other hobbies. (2) The inhabitants operate the club. (3) It can be operated without financial support from the local governments. (4) This is repeated monthly, at a minimum.

The “*Kayoinoba*” in A City is commonly held for about one hour at one time. There were 30–40 participants at a time. The program’s content was mainly an exercise, as the participants watched a video tape recording of simple gymnastics. Following the progression of COVID-19, various infection control measures were conducted: measurements of body temperature, wearing face masks, washing hands, being in well-ventilated areas, shortening of hours, and participants’ needing to keep quiet before and after the exercise. During the state of emergency and the occurrence of the COVID-19 clustering near the “*Kayoinoba*,” the activities were completely stopped. During the periods other than those mentioned above, “*Kayoinoba*” was active.

### 2.3. Evaluation of Mental and Physical Function and Frailty

The Kihon Checklist (KCL) has been used in clinical practice in Japan to evaluate the mental and physical functions and to diagnose frailty in the elderly. The KCL was developed as a screening tool. Here, the public health nurses assigned to the CGSC can detect high-risk elderly individuals at an early stage [17,18,19]. The KCL has been validated using the Cardiovascular Health Study (CHS) frailty index by Fried et al. [20]. This is the most acceptable instrument to diagnose frailty among the elderly [21,22].

KCL consists of 25 question items. Either “Yes” or “No” was selected for each question’s item. The 25 items were classified into seven domains (Table 1): Lifestyle of daily living by 20 items (#1–20); physical function by five items (#6–10); nutrition by two items (#11–12); oral function by three items (#13–15); socialization by two items (#16,17); cognitive function by three items (#18–20) and depressive mood by five items (#21–25). Each cut-off point was used to discriminate between the absence or presence of risk in each domain.

The changes in each domain’s status from the baseline to a one-year follow-up were compared and three types were classified: “Worsened” is the case from “without the risk” to “with the risk;” “Unchanged” from “without the risk” to “without the risk” or from “with the risk” to “with the risk;” “Improved” from “with the risk” to “without the risk.” According to the report by Satake et al. [21], scores of ≥8 points and scores of <8 points in all 25 items of KCL were defined as “frailty” and “robust,” respectively.

### 2.4. Survey Time and Method

**Figure 2:**
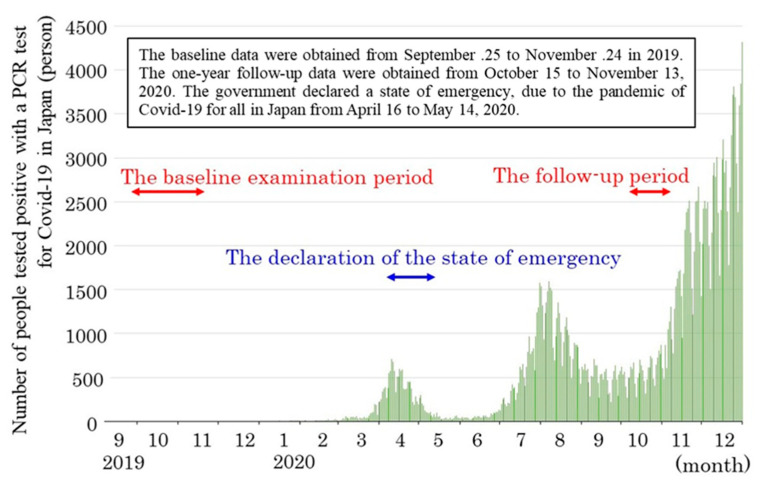
The number of COVID-19 cases in Japan and sampling times.

Figure 2 shows the number of COVID-19 infections in Japan and the sampling times in this study. The recording period for the baseline was before the COVID-19 pandemic, between 25 September 2019 and 24 November 2019. The recording period for the one-year follow-up was after the first increase of COVID-19 in Japan and at the time of re-increase between 15 October 2020 and 13 November 2020.

The detention method was applied for the survey. Thus, public health nurses left the questionnaire sheets at the respondent’s home after obtaining their informed consent from the questionnaire survey and picked them up after a certain period. Generally, the recoding of KCL is a standard process, whereby the nurses directly ask the questions to each participant using a KCL sheet. However, it was changed to self-administration due to the spread of COVID-19.

Personal attributes were assessed by multiple-choice: age (65–74 years old/ over 75 years old), gender (male/ female), living style (living alone/couples/with children), affluence for living (affluence/normal/non affluence), and the frequency of participation in “*Kayoinoba*” (everyday/once a week/once a month/once a year). The answers at the baseline were used as representative of each respondent. Those at the one-year follow-up were used to complete missing values at the baseline.

### 2.5. Statistical Analysis

For the statistical analysis, the McNemar test, Chi-square test, and Haberman’s residual analysis were used to compare the changes in the values between the baseline and the one-year follow-up. The adjusted residual values were evaluated with the standard normal deviation value of 1.96 at a significance level of 0.05 [23]. All the statistical analyses were conducted at a significance level of 0.05. This was done by using the Statistical Package for the Social Sciences (SPSS^®®^) software (version 24.0, IBM Corp., Armonk, NY, USA).

In this study, missing values were found in 25 items of the KCL, since the self-administration process of data collection was used. It was reported that the exclusion of respondents with missing values causes selection bias [24]. The missing values were supplemented by using the multiple imputation method, based on the iterative Monte Carlo method using SPSS^®®^ Missing Values version 24.0 (IBM Corp. Armonk, NY, USA).

The survey was conducted with the approval of the Tokushima University Hospital Medical Research Ethics Review Committee (3250-1). A City collected the questionnaires for public health work and the anonymized data were used as secondary data in this study.

## 3. Results

The questionnaire collection rate was 100%. Both the baseline and one-year follow-up surveys initially targeted 136 respondents. No deaths or withdrawals due to hospitalization were observed. At the baseline and one-year follow-up, 80 respondents had missing values for any one of the KCL question items. Therefore, 15 individuals who had missing values of more than two items (12% or more of all items) in 25 items were excluded from the analysis. In addition, 20 respondents who had missing values for the frequency of participation in “*Kayoinoba*,” were excluded. Finally, 101 respondents were used for the final analysis. The missing values were supplemented with KCL responses. The percentage of missing values for each item ranged from 0% to 5.0% for both the baseline and one-year follow-up and accounted for 1.1% of the total.

Table 2 shows the personal properties of the 101 study participants. The sex ratio was 85 females (84.2%) and 16 males (15.8%). There were 37 (36.6%) respondents aged 65–74 years and 64 (63.4%) aged 75 and over. Twenty-two respondents lived alone (21.8%), 35 lived as couples (34.7%), and 44 respondents lived with their children (43.5%). The number of respondents according to affluence, normal, and non-affluence were eight (27.7%), 18 (17.8%), and 55 (54.5%), respectively.

Table 3 shows the changes in the frequency of the participation in “*Kayoinoba*” between the baseline and one-year follow-up. Two respondents were classified as “every day” (2.0%), 64 respondents (63.4%) as “once a week,” and 35 respondents (34.6%) as “once a month,” at the baseline. There were three respondents (3.0%) classified as “every day,” 68 respondents (67.3%) as “once a week,” 29 respondents (28.7%) as “once a month,” and one respondent (1.0%) as “once a year” at the one-year follow-up. There was no difference in the participation status before and after the spread of COVID-19.

Table 4 shows the distributions of the three types of changes: “Improved,” “Unchanged,” and “Worsened” in the seven domains of KCL from the baseline to the one-year follow-up. The Chi-square test revealed a significant association between the distribution of change in the seven domains of KCL between the baseline and one-year follow-up. The “unchanged” was the highest in all seven domains of KCL, especially in the nutritional status, which was 100%. The “improved” was higher in the following domains: oral function, socialization, cognitive function, and having a depressed mood. The numbers of “improved” and “worsened” were the same in the domains of lifestyle and physical functioning. As a result of the adjusted residuals, the ratio of “worsened” in their physical activity was significantly higher than those in other domains. Conversely, that in the nutritional status was significantly lower. In addition, there were few participants with a low BMI of less than 18.5. Of 101 total participants, six persons had low BMI at the baseline and four at the one-year follow-up.

Table 5 shows the ratios of “robust” and “frailty” between the baseline and one-year follow-up. As a result of the McNemar test, no significant difference was found in the ratio between the baseline and one-year follow-up (*p*-value = 0.424). From the analysis of the adjusted residuals, the unchanged extent of the robust/frailty groups in the two periods was significantly higher than the changed extent. The ratio of frailty tended to decrease from 23.8% to 19.8% between the baseline and one-year follow-up.

## 4. Discussion

This study aimed to clarify how “*Kayoinoba*,” which is one of the preventive measures against frailty in Japan, was operated under self-quarantine, due to the spread of COVID-19. It shows how the mental and physical functions of the elderly were maintained, as a result. The survey area was conducted in a standard rural area in Japan, with many late-stage female elderly. There were few participants with a low BMI and a low COVID-19 infection rate, including 37 SARS-CoV-2 positive persons in August 2020, eight persons in September 2020, and none since then. It is necessary to discuss the results of this study while considering such regional characteristics.

Under self-quarantine, due to the spread of COVID-19, while “*Kayoinoba*” has been operated, the number of participants did not decrease between the baseline and one-year follow-up. The frequency of the participants’ participation was also maintained. At the one-year follow-up, the number of COVID-19 cases increased. However, the state of emergency had already been lifted six months prior, and the activity of “*Kayoinoba*” was resumed. Through the interviews with the CGSC staff, it was confirmed that the spread of COVID-19 resulted in the decrease in people exchange and an increase in the feeling of loneliness. They had also alerted the residents to the fact that the participation in “*Kayoinoba*” reduced isolation before the COVID-19 pandemic. Therefore, daily educational activity may lead to increased participation by the elderly in “*Kayoinoba*,” during the spread of COVID-19, through its appropriate operation. In addition, the changes in cognitive function and depressive mood in KCL during the baseline and one-year follow-up showed that the ratio of “Unchanged” was the highest followed by “Improved.” Although previous studies reported on the mental health deterioration of the elderly, such as an increase of anxiety and depression due to the spread of COVID-19 [4,5,6], the results of this study suggest that mental health was comparatively maintained or improved. The main purpose of “*Kayoinoba*” is to provide a place for exercise to maintain physical function, but it can also be interpreted as a place for human interaction, and so it contributed to the reduction of anxiety and the elimination of depressive mood for the elderly.

Regarding the changes in each domain’s status of KCL from the baseline to the one-year follow-up, the ratios of “Unchanged” were the highest in every domain. The mental and physical functions of the elderly did not decrease, even under the conditions of self-quarantine during COVID-19. In addition, the ratio of “Worsened” in the domain of physical activity was significantly higher than that of the other domains. Conversely, the ratio was significantly lower in the domain of nutritional status. This may be due to the fact that “*Kayoinoba*” was temporarily interrupted due to the spread of COVID-19, and that the hours were shortened after the resumption. Kitamura et al. [25] showed that “*Kayoinoba*” in A City was exercise-oriented in a comparative study focusing on the “*Kayoinoba*” program. Therefore, the interruption of “*Kayoinoba*” due to self-quarantine may reduce physical functioning, as the exercise habits of the elderly were established by their participation in “*Kayoinoba*.” Conversely, the nutritional status was maintained because meals could be regularly served at home during the self-quarantine period as indicated by the decrease in participants with low BMI from six to four.

The rates of the weakened respondents at the baseline and one-year follow-up, that were 23.8% and 19.8%, respectively, were higher than the values of 8.7% and 11.3% reported by Murayama [13] and Shimada [14] before the COVID-19 pandemic. In addition, Ohashi et al. [26] analyzed 551 home-based elderly people between 65 and 70 years of age (excluding the dropouts from the 1053 respondents) for five years, according to the same method as in this study. They also reported that the ratio of “worsened” was significantly higher in the domains of physical activity and oral functioning than those in other domains, and that nutrition, socialization, and cognitive function were significantly lower. Although Ohashi’s study was a cohort study of a natural follow-up for five years, the results were similar to those of the present study under self-quarantine, due to the spread of COVID-19. In addition, the rate of frailty in Ohashi’s study increased from 8.0% to 12.3% over five years. The rate of frailty tended to increase over time. Conversely, the rate of frailty at the one-year follow-up in this study decreased when compared to that at the baseline, although both rates during the two periods were higher. This is because the spread of COVID-19 was minimal, such that “*Kayoinoba*” worked to suppress the deterioration of mental and physical functions of the elderly and to prevent the progress of frailty.

Although no significant factor of the basic attributes that influence the rate of frailty could be found in the regression analysis, the rate of frailty in family living tended to be smaller than in single living conditions. This may mean that the “*Kayoinoba*” is significant in preventing frailty in the elderly who live alone. In addition, the CGSC staff has repeatedly informed the resident elderly through the activities of “*Kayoinoba*” that frailty is a predictor of death and long-term care. It is assumed that the respondents are fully aware of the risk of frailty. Kulmala et al. [27] reported that lifestyle and exercise habits influence frailty in the long-term. In addition, Peterson et al. [28] showed a relationship between the amount of activity in daily life and the occurrence of frailty. This suggested that daily active prevention of frailty is important. It is important to show the efficacy of “*Kayoinoba*” and its improvement measures with direct evidence.

This study has three major limitations. First, KCL was originally evaluated by professionals, such as public health nurses asking the elderly the question items in a face-to-face manner. However, at this time, it was conducted via a self-administered method for the elderly in their self-quarantine, due to COVID-19. Consequently, the responses have some missing values, and the supplementation of these values may cause a decline in the data’s reliability. The highest missing rate for one question item was 5%. The overall average missing rate was 1.1%. The sample size was 101 participants. Demirtas et al. [29] and Graham et al. [30] state that the criteria of a reliable supplement include that the missing rate is less than 25% and that the sample size is more than 100. The data acquired in this study sufficiently met these criteria, and the supplement was reliable. Second, the selection of respondents was based on the CGSC public health nurses’ ties. The results may thus demonstrate a sampling bias and regional idiosyncrasies. Third, the KCL does not always directly show the elderly’s mental and physical functions and their state of frailty. A more direct assessment will be required to show the significance of “*Kayoinoba*” with direct evidence.

## 5. Conclusions

It is suggested that the participants in “*Kayoinoba*” may have suppressed the deterioration of mental and physical conditions, excluding their physical activity. It appears that it may have assisted in preventing frailty among the elderly, even during their self-quarantine, due to the spread of COVID-19. “*Kayoinoba*,” a place for human exchange among home-based elderly, is likely to have contributed to the reduction of anxiety and loneliness and the elimination of depressive mood. Further activities to establish the concept of frailty and “*Kayoinoba*” in the resident elderly should be continued.

## Figures and Tables

**Table 1:** Kihon Checklist (top) and its seven domains (bottom).

**No.**	**Question**	**Answer**	
1	Do you go out by bus or train by yourself?	0. Yes	1. No
2	Do you go shopping to buy your daily necessities by yourself?	0. Yes	1. No
3	Do you manage your own deposits and savings at the bank?	0. Yes	1. No
4	Do you sometimes visit your friends?	0. Yes	1. No
5	Do you tum to your family or friends for advice?	0. Yes	1. No
6	Do you normally climb stairs without using handrails or walls for support?	0. Yes	1. No
7	Do you normally stand up from a chair without any aids?	0. Yes	1. No
8	Do you normally walk continuously for 15 min?	0. Yes	1. No
9	Have you experienced a fall in the past year?	1. Yes	0. No
10	Do you have a fear of falling while walking?	1. Yes	0. No
11	Have you lost 2 kg or more in the past 6 months?	1. Yes	0. No
12	Height: cm, weight: kg, BMI: kg/m² If BMI is less than 18.5, this item is scored	1. Yes	0. No
13	Do you have any difficulties eating tough foods compared to 6 months ago?	1. Yes	0. No
14	Have you choked on your tea or soup recently?	1. Yes	0. No
15	Do you often experience having a dry mouth?	1. Yes	0. No
16	Do you go out at least once a week?	0. Yes	1. No
17	Do you go out less frequently compared to last year?	1. Yes	0. No
18	Do your family or your friends point out your memory loss? E.g., “You always ask the same question over and over again.”	1. Yes	0. No
19	Do you make a call by looking up phone numbers?	0. Yes	1. No
20	Do you find yourself not knowing today’s date?	1. Yes	0. No
21	In the last two weeks have you felt a lack of fulfilment in your daily life?	1. Yes	0. No
22	In the last two weeks have you felt a lack of joy when doing the things you used to enjoy?	1. Yes	0. No
23	In the last two weeks have you felt any difficulty in doing what you could do easily before?	1. Yes	0. No
24	In the last two weeks have you felt helpless?	1. Yes	0. No
25	In the last two weeks have you felt tired without a reason?	1. Yes	0. No
**Domains**	**Relevant Questions**	**Cut-Off Point with/without Each Risk**	
Lifestyle	#1–20	Ten or more negative answers	
Physical function	#6–10	Three or more negative answers	
Nutrition	#11, #12	Negative answers to both questions	
Oral function	#13, #14, #15	Two or more negative answers	
Socialization	#16, #17	An answer of “No” to #16	
Cognitive function	#18, #19, #20	One or more negative answers	
Depressive mood	#21–25	Two or more negative answers	

**Table 2:** Characteristics of the study sample.

Attributes	Numbers (%)
Sex	
Females	85 (84.2)
Males	16 (15.8)
Age (ys.)	
65–74	37 (36.6)
≥75	64 (63.4)
Living style	
Alone	22 (21.8)
Couple	35 (34.7)
with child/children	44 (43.5)
Affluence for living	
Affluence	28 (27.7)
Normal	18 (17.8)
Non affluence	55 (54.5)

**Table 3:** Comparison of the participation frequency between the baseline and one-year follow-up.

	Everyday	Once a Week	Once a Month	Once a Year
Baseline	2 (2.0)	64 (63.4)	35 (34.6)	0 (0.0)
One-year follow-up	3 (3.0)	68 (67.3)	29 (28.7)	1 (1.0)

Number of relevant persons (%).

**Table 4:** Comparisons of the seven domains in the Kihon Checklist between the baseline and one -year follow-up.

	Improved	Unchanged	Worsened	Adjusted Residuals for Worsened Transition
Lifestyle	5 (5.0)	91 (90.0)	5 (5.0)	−0.90
Physical function	13 (12.9)	75 (74.2)	13 (12.9)	2.50 *
Nutrition	0 (0)	101 (100)	0 (0)	−3.00 *
Oral function	11 (10.9)	81 (80.2)	9 (8.9)	0.80
Socialization	4 (4.0)	94 (93.0)	3 (3.0)	−1.70
Cognitive function	19 (18.8)	71 (70.3)	11 (10.9)	1.60
Depressive mood	15 (14.9)	77 (76.2)	9 (8.9)	0.80

Number of relevant persons (%). Chi-square test * Statistically significant at the level of *p* < 0.05.

**Table 5:** Comparisons of Robust/Frailty status between the baseline and one-year follow-up.

		One-Year Follow-Up	
		Robust	Frailty
Baseline	Robust	72 (71.3)/6.0 *	5 (5.0)/−6.0 *
	Frailty	9 (8.9)/−6.0 *	15 (14.9)/6.0 *

Top value: the relevant person’s numerical identifier, bottom value: (%)/adjusted residuals, McNemar test. * Statistically significant at the level of *p* < 0.05.
